# Quantitative Response of Gray-Level Co-Occurrence Matrix Texture Features to the Salinity of Cracked Soda Saline–Alkali Soil

**DOI:** 10.3390/ijerph19116556

**Published:** 2022-05-27

**Authors:** Yue Zhao, Zhuopeng Zhang, Honglei Zhu, Jianhua Ren

**Affiliations:** 1 Heilongjiang Province Key Laboratory of Geographical Environment Monitoring and Spatial Information Service in Cold Regions, Harbin Normal University, Harbin 150025, China; zhaoyue_hrbnu@163.com (Y.Z.); zhuopeng_hrbnu@163.com (Z.Z.); 2 College of Life Science, Henan Normal University, Xinxiang 453007, China; 2015111@htu.edu.cn

**Keywords:** GLCM, texture feature, soda saline–alkali soil, soil surface crack, Songnen Plain

## Abstract

Desiccation cracking during water evaporation is a common phenomenon in soda saline–alkali soils and is mainly determined by soil salinity. Therefore, quantitative measurement of the surface cracking status of soda saline–alkali soils is highly significant in different applications. Texture features can help to determine the mechanical properties of soda saline–alkali soils, thus improving the understanding of the mechanism of desiccation cracking in saline–alkali soils. This study aims to provide a new standard describing the surface cracking conditions of soda saline–alkali soil on the basis of gray-level co-occurrence matrix (GLCM) texture analysis and to quantitatively study the responses of GLCM texture features to soil salinity. To achieve this, images of 200 field soil samples with different surface cracks were processed and calculated for GLCMs under different parameters, including directions, gray levels, and step sizes. Subsequently, correlation analysis was then conducted between texture features and electrical conductivity (EC) values. The results indicated that direction had little effect on the GLCM texture features, and that four selected texture features, contrast (CON), angular second moment (ASM), entropy (ENT), and homogeneity (HOM), were the most correlated with EC under a gray level of 2 and step size of 1 pixel. The results also showed that logarithmic models can be used to accurately describe the relationships between EC values and GLCM texture features of soda saline–alkali soils in the Songnen Plain of China, with calibration R^2^ ranging from 0.88 to 0.92, and RMSE from 2.12 × 10^−4^ to 9.68 × 10^−3^, respectively. This study can therefore enhance the understanding of desiccation cracking of salt-affected soil to a certain extent and can also help to improve the detection accuracy of soil salinity.

## 1. Introduction

Soil salinization is a very serious issue in China, with the total area of salt-affected soil being almost 9.91 × 10^7^ ha [1], which is increasing due to the growing population and deteriorating ecological environment. This has caused great damage to China’s social economy, natural environment, and ecosystem. As one of the three major distribution areas in China, the total area of saline soil in the Songnen Plain is over 3.73 × 10^6^ ha [2]. The main soil salt minerals in this area are NaHCO_3_ (sodium bicarbonate) and Na_2_CO_3_ (sodium carbonate), together with small amounts of sulfate and chloride, indicating that the salt-affected soils belong to a typical type of inland soda saline–alkali soil. Because of the high content of clay particles and adsorbable cations, shrinkage and cracking on the surface of soda saline–alkali soil are very common during water evaporation. Desiccation cracks indicate a surface state of saline–alkali soils and are commonly considered as a characterization of the salinity levels of salt-affected soils, which also indicate a mechanical state of the salt-affected soil [3]. Therefore, exploring the quantitative relationship between crack characteristics and salt content will help to further understand the cracking mechanism of the saline–alkali soil surface. In addition, effective crack characteristics can also be used to further predict soil salinity in order to provide important guidance for both ecological restoration and improvement of salinized soil in soil sciences, which will thus alleviate the conflicts between human and local land and will thus guarantee China’s food security to a certain extent.

Many field and laboratory studies have focused on the relationship between soil salinity and the morphology of desiccation cracks. Amaya et al. [4] measured salinity of a saline cracking soil within the peds to a depth of 50 cm over a three-year period during reclamation drying in field, finding that although the initial EC within the interior of peds and below the cracking depth ranged from 22 to 35 ds/m, the soil salinity redistributes and decreases with the EC range from only 12 to 16 ds/m at the end of three years of desalinization. Lima and Grismer [5] conducted field measurements considering the effects of soil salinity on soil crack morphology at various times after irrigation and found that as salinity increased, soil crack width, island width, crack area, and crack volume tended to increase, whereas crack depth decreased. In order to present a field dataset to quantitatively evaluate the contribution of bypass flow to the leaching salts, Fujimaki and Baki [6] carried out soil sampling and monitoring of groundwater and discharge from a tile drain in farmland with a cracking soil in the Nile Delta, finding that the evidence for the occurrence of significant bypass flow through cracks was the salinity of the pore water. After conducting a field evaluation of the impact of soil cracking on irrigation, drainage, and soil salinity on a heavy clay soil in the Imperial Valley of California, Van der Tak and Grismer [7] found that water movement within soil cracks controls the water application uniformity, soil profile wetting, and salt leaching to irrigation. Ben-Hur and Assouline [8] selected a cotton field located in the Yizre’el Valley, Israel, as the experiment site to study the tillage effects on water and salt distribution in a vertisol during effluent irrigation and rainfall, with their results showing that the high infiltration of the runoff through cracks limited the effects of the runoff downhill flow on the water and salt distribution along the slope. After studying the effects of roots and salinity on law of development for farmland soil desiccation crack, Zhang et al. [9] found that when the moisture content is less than 27%, salt content will greatly increase the area density of soil cracks at steady state with the length density appearing as a rather opposite trend. However, most field experiments only deal with the qualitative relationship between salt content and desiccation cracks. With the development of image processing technology, crack feature can be extracted with a quite precise accuracy, which makes more scholars tend to carry out controllable experiments to quantitatively analyze the influence of salt content on soil desiccation cracking. From a laboratory dry test, Zhang et al. [10] investigated the development law of desiccation cracks on the soil surface under different salt content, with their results indicating that soil salinity can increase area density but lead to a decrease in length density of cracks. Zhang et al. [11] conducted desiccation tests in the laboratory on initially saturated slurry specimens with different NaCl (sodium chloride) content selected from Yar City, northwest China, and their results indicated that as the NaCl content increased, the intersection number, segment number, and total length of the cracks all decreased. After conducting a laboratory study to investigate the effects of four salt cations, namely, Na^+^ (sodium), K^+^ (potassium), Ca^2+^ (calcium), and Mg^2+^ (magnesium), on soil shrinkage and cracking during dehydration, Xing et al. [12] found that parameters including crack length, crack area, crack length density, and crack area density decreased with an increase in the concentrations of K^+^, Na^+^, and Ca^2+^, but a reduction was found when the concentration of Mg^2+^ increased, indicating that the four crack parameters also increased with the content of HCO_3_^−^ (bicarbonate), CO_3_^2−^ (carbonate), and SO_4_^2−^ (sulfate), while an opposite trend was found with the concentration of Cl-. After studying the effect of cation type on the process of shrinkage and desiccation cracking, Wang et al. [13] found that salt cations, including Na^+^, K^+^, Ca^2+^, and Mg^2+^, have a strong effect on the crack areas of salt-affected soils. 

From the studies mentioned above, it can be seen that the relationships between salt content and the extent of desiccation cracks are quite different, indicating that the effect of salinity on the cracking patterns and dynamics of salt-affected soils still remains an open question. This is because of the complicated interaction between salt and soil particles during desiccation cracking is affected by the ion concentration and the valence state of ions, which are determined by the physical and chemical properties of the soil samples, indicating that the effect of salinity on the cracking of the cohesive soil surface remains partially unclear [14]. In addition, the response of soil cracking to salt content is inseparable from the selection of crack characterization indicators. Although many quantitative indexes in previous studies have been established to characterize the extent of shrinkage and desiccation cracks [15,16,17,18], there is still no unified soil crack description standard. In addition, most previous indicators have focused on geometric features, indicating that they usually have severe limitations for characterizing cracks and cannot reflect the spatial distributions of desiccation cracks with convergence in many applications. Moreover, these geometric indicators hardly reflect the direction of crack propagation, which usually depends on the local hydrological conditions and topographical fluctuations of salinized soil. Therefore, an effective characterization indicator of soil cracks is of great significance for analyzing the response of crack characteristics to soil salt content, determining the salt migration process of the saline–alkaline soil, monitoring the range of soil salinization, and ameliorating salt-affected soil.

Texture features often refer to visual characteristics that do not depend on the color or brightness of the image and can reflect the homogeneous phenomenon of the image and describe the pixel distribution in the neighborhood space [19,20,21]. For a special object within an image, texture features often contain important information about the surface structure arrangement and thus can reflect its connection with the surrounding environment [22,23]. Texture analysis aims to select a unique method to describe the underlying characteristics, which generally consist of four types: statistical, modeling, signal processing, and structural methods [24]. Among these types of texture analysis, GLCM is the most common [25,26,27,28,29,30] method because it can reflect a large amount of information within a grayscale image, such as the direction, interval, amplitude, and change ratio. GLCM texture features are commonly extracted for analyzing the local features and overall arrangement rules of an image and are widely used for pattern recognition, accurate classification, feature extraction, and image segmentation in many applications [31,32,33,34,35,36,37,38,39]. Because the propagation and development of desiccation cracks are rather random in statistics, texture features always contain important information about the crack arrangement and the pixel distributions within the crack patterns. Therefore, it is quite certain that GLCM texture analysis can aid in describing the complex structures and variation in the surface intensity of desiccation cracks in cohesive saline–alkali soil. This is because GLCM texture features can describe both the important arrangement of the surface structure and the distribution of the pixels of the soil crack image in the neighborhood space. In addition, GLCM texture features extracted from crack patterns can also reflect differences in the physical and chemical properties of saline–alkali soils, such as clay minerals and salt content. However, very few studies have focused on the relationship between soil salinity and GLCM texture features computed from crack images. However, research on the correlation between cracked soil surface texture features and soil salinity is still very rare. Although Ren et al. [40,41] studied the influence of salt content on the shrinkage and cracking process of soda saline–alkali soils on the basis of the theory of GLCM texture analysis, only one type of GLCM texture feature (corresponding to the contrast) was extracted from the binary crack image in their research, and their cracked soil samples were prepared in the laboratory, indicating that these samples cannot accurately reflect the real status of desiccation cracks generated in nature. 

Surface cracking is a mechanical state of saline–alkali soil, which indicates that exploring the correlation between crack characteristics and salt content can therefore effectively improve the cognition level of cracking process of saline soil. To achieve this objective and develop an effective standard for crack characteristics, this study intended to quantify the ability of GLCM texture features extracted from crack patterns in characterizing the desiccation cracks generated on the surface of soda saline–alkali soil samples. In particular, the effects of parameters, including the gray level, step size, and direction, on the results of GLCM texture features were individually analyzed and compared. Subsequently, correlation analysis was also conducted between GLCM texture features and EC values of the soil samples to quantitatively study the response of GLCM texture features to soil salinity for further understanding the cracking process of soda saline–alkali soils in the Songnen Plain of China. Finally, the logarithmic regression model between EC values and several common texture features were developed under the optimal GLCM computing parameters, which both leads to a better recognition for desiccation cracking process of soda saline–alkali soils and also provides a possibility for effective detection of the characteristics of soda saline–alkali soils.

## 2. Materials and Methods

### 2.1. Study Area and Soil Sampling

**Figure 1:**
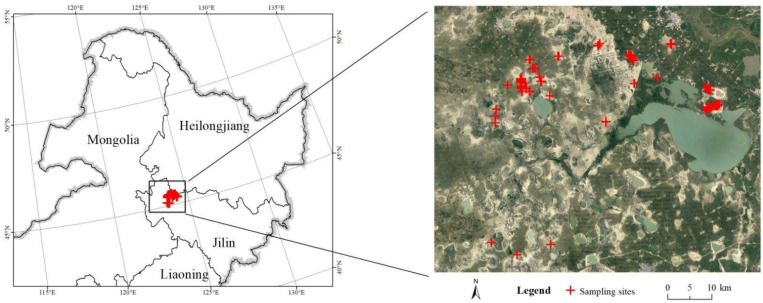
This study area and sampling points located on a Landsat-8 satellite image.

The western part of the Songnen Plain is a typical salt-affected soil region in China with salt minerals mainly composed of NaHCO_3_ and Na_2_CO_3_, which makes the soil a typical type of soda saline–alkaline soil. In this study, Baicheng City was selected as the study area, with an average annual precipitation of 400 to 500 mm, which is mainly concentrated in July and August; however, the average annual evaporation in Baicheng City is as high as 1500 to 1900 mm. This severely unbalanced evaporation-to-precipitation ratio, coupled with special hydrogeological conditions and over-irrigation in human agricultural production, makes the area heavily salinized. In addition, the desiccation cracks commonly occur on the soil surface since the soil can be classified as a texture of clay loam with high clay content. After considering the heterogeneity of soil salinity, 200 soil sample points with different extents of desiccation cracking were selected, with a small region ranging from 45°18′14′’ N to 45°29′47′’ N and 123°39′8′’ E to 124°21′6′’ E in November 2018 (Figure 1). This is because, after months of evaporation, the soil moisture content is very low in November, and the cracking process is complete on the soil surface. All soil samples were determined on the basis of the rule of plum blossom spots.

### 2.2. Preprocessing of Crack Images

**Figure 2:**
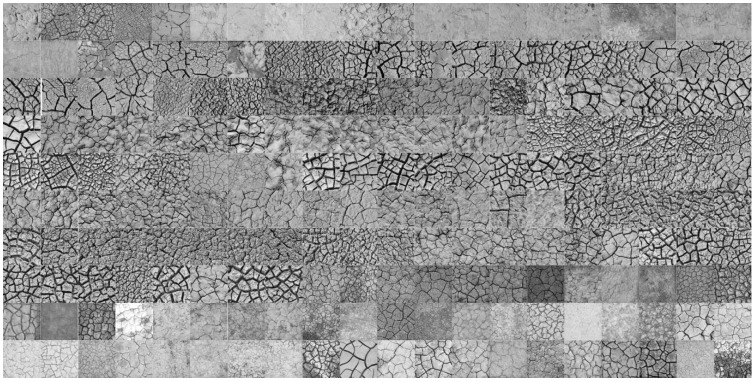
Grayscale images of cracked soil samples.

According to Ren et al. [40], the GLCM contrast feature becomes stable at a scale of approximately 38 × 38 cm. After considering the effects of sample size on the degree of soil cracking, soil samples with a rectangular size of 50 × 50 cm were selected in this study, and the cracking patterns of all the soil samples were measured using the following standard. First, a digital camera was selected and fixed on a platform with the lens 1.5 m above the ground. Second, a rectangular wooden frame with an inner size of 50 × 50 cm was designed and placed on the ground to ensure that it coincided with the vertical projection of the digital camera lens. Third, the white balance processing of the camera was performed together with the aperture size and exposure time set to standardize the same lighting environment and camera parameters. Finally, the desiccation crack was photographed and the calibration image of a black and white grid plate with a size of 50 × 50 cm was taken for further geometric distortion corrections. When the images of the cracked soil samples were taken, a unified pre-processing operation was conducted. In detail, all crack patterns of the soil samples were geometrically corrected using the polynomial method, cropped to a standard size of 50 × 50 cm, and converted into grayscale images (Figure 2). 

### 2.3. Soil Property Measurements

**Figure 3:**
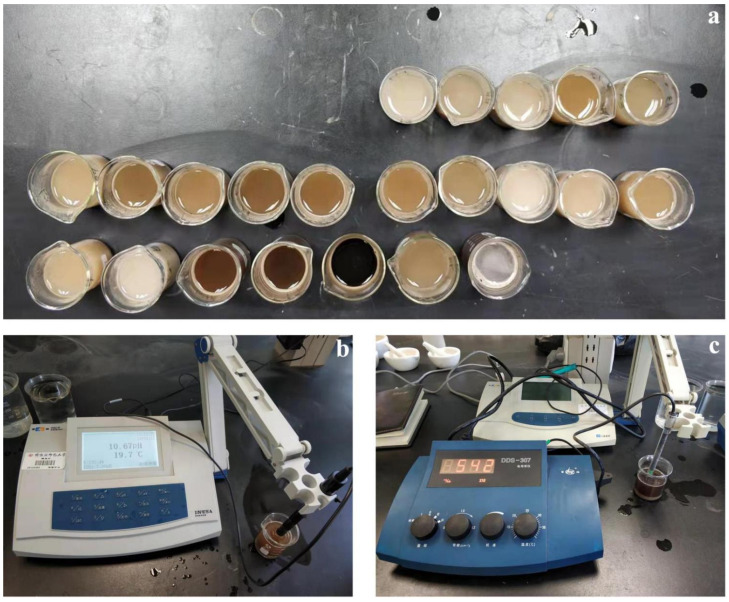
Measurements of main chemical soil properties. (**a**) Prepared soil suspensions; (**b**) pH measurement process; (**c**) EC measurement process.

After the crack images were taken at the sampling points, soil samples were collected at a depth of 20 cm in the center of the wooden frame to determine the soil properties. This is because the kind of soda saline soil largely prevents salt moving downwards due to its bad infiltration capacity, which indicates the properties of soil from the top 20 cm soil layer are very stable [42]. The soil samples were weighed before and after they were evenly oven-dried for water contents, after which they were ground and passed through a 2 mm sieve for soil properties. Note that as the osmotic pressure of the soil solution increased by soil salinity is strictly proportional to the EC values under certain water conditions, the EC value was thus determined as the indicator of soil salinity in this study. In particular, soil suspensions for all soil samples were configured using CO_2_-free distilled water (pH = 7), with a water–soil mass ratio of 5:1 [43,44,45]. After stirring with a glass rod and leaving for about half an hour, the pH and EC values of all the soil samples were measured using potentiometric and conductometric methods, respectively (Figure 3). To facilitate the analysis and comparison of different samples, the unit of the EC value was uniformly converted into ds/m. In addition, the particle size distributions of all soil samples were measured using an Mllvern-200 laser particle size analyzer. 

### 2.4. Gray Level Co-Occurrence Matrix Texture Features

In this study, we selected the classic GLCM texture features as the statistical texture characteristics of the cracked soil surface to determine the relevance of different pixels by calculating the second-order combined conditional probability density between the image pixel gray levels at a certain distance and direction. For a known gray image *f*(*x*,*y*) of a soil sample with surface cracks, the second-order combined conditional probability density can be calculated using the following equation:

(1) $$p\left(i,j\right)=g\left\{\left(x_{1},y_{1}\right),\left(x_{2},y_{2}\right)\in m\times n|f\left(x_{1},y_{1}\right)=i,f\left(x_{2},y_{2}\right)=j\right\}$$

where *i* and *j* are the gray values of the gray image *f*(*x*, *y*) at the (*x*_1_, *y*_1_) and (*x*_2_, *y*_2_) coordinate positions in Equation (1), respectively. In practical applications, a series of texture feature features must be extracted according to the calculation results of the gray-level co-occurrence matrix so that the images can be more intuitively extracted and target recognitions can be performed. After the GLCMs of all cracked soil samples were extracted, 13 common texture features were then computed using the formulas listed in Table 1 [46].

Here, p(i,j) is the value of the normalized GLCM at the (i,j) coordinate position; Ng is the gray level of the co-occurrence matrix; $u_{x}$ and $u_{y}$ are the mean of $p_{x}\left(i\right)$ and$\;p_{y}\left(j\right)$, respectively; and $\sigma_{x}\;$and $\sigma_{y}\;$are the variance of $p_{x}\left(i\right)$ and$\;p_{y}\left(j\right)$, respectively. The relevant intermediate parameters in the formulas for texture features can be calculated from the following Equations (2)–(7):

(2) $$\;\;\;\;\;\;\;\;\;\;P_{x}\left(i\right)=\sum_{j=1}^{Ng}p\left(i,j\right)$$

(3) $$\;\;\;P_{y}\left(j\right)=\sum_{i=1}^{Ng}p\left(i,j\right)$$

(4) $$P_{x+y}\left(k\right)=\overset{Ng}{\underset{i=1}{\sum }}\overset{Ng}{\underset{j=1}{\sum }}p\left(i,j\right),i+j=k,k=2,3,\ldots 2Ng$$

(5) $$HXY=-\overset{Ng}{\underset{i=1}{\sum }}\overset{Ng}{\underset{j=1}{\sum }}p\left(i,j\right)log\left\{p\left(i,j\right)\right\}$$

(6) $$HXY1=-\overset{Ng}{\underset{i=1}{\sum }}\overset{Ng}{\underset{j=1}{\sum }}p\left(i,j\right)log\left\{p_{x}\left(i\right)p_{y}\left(j\right)\right\}$$

(7) $$\;\;\;\;\;\;\;\;\;\;HXY2=-\overset{Ng}{\underset{i=1}{\sum }}\overset{Ng}{\underset{j=1}{\sum }}p_{x}\left(i\right)p_{y}\left(j\right)log\left\{p_{x}\left(i\right)p_{y}\left(j\right)\right\}$$

**Figure 4:**
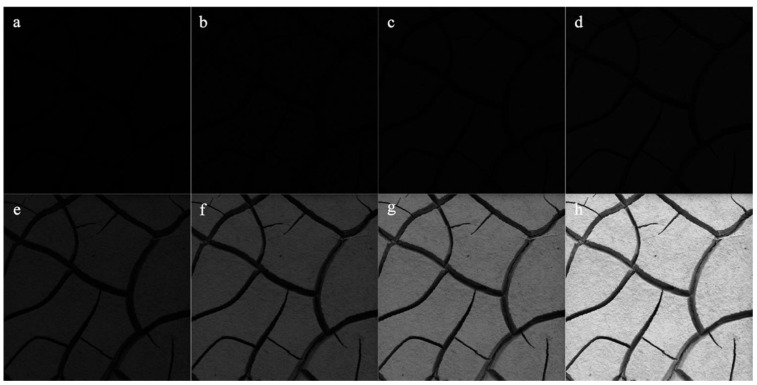
Extraction results of eight gray-scale images of a typical cracked soil sample. (**a**) gray level of 2; (**b**) gray level of 4; (**c**) gray level of 8; (**d**) gray level of 16; (**e**) gray level of 32; (**f**) gray level of 64; (**g**) gray level of 128; (**h**) gray level of 256.

To study the influence of GLCM calculation parameters on the results of texture features, the crack images of all soil samples were transformed into eight different gray levels (2, 4, 8, 16, 32, 64, 128, and 256, as shown in Figure 4), and the GLCMs of the transformed images with different gray levels were calculated at seven different pixel steps (1, 5, 10, 20, 40, 60, and 80 pixels) and four directions (0°, 45°, 90°, and 135°) for texture features. Correlation analysis was then performed between EC and texture features on the basis of different GLCM parameters. 

Note that there were too many soil samples in this study, indicating that different types of measurement results cannot be listed individually. In order to express the characteristics of the data more clearly, statistical analysis was therefore performed on the measured physical and chemical properties of soil samples and the GLCM texture features extracted from crack images. Specially, the statistical parameters including the minimum, maximum, average, standard deviation, coefficient of variance (CV), skewness, and kurtosis values of soil properties and GLCM texture features were computed using the SPSS software.

## 3. Results

### 3.1. Chemical and Physical Properties

Table 2 lists the statistical indexes of soil properties for all the cracked soil samples. From the table, it can be seen that EC values of the samples ranged from 0.2 to 6.37 ds/m with a mean value of 0.95 ds/m, while pH ranged from 8.55 to 11.16 with a mean value of 10.06. In addition, measurement results from Li and Wang [47] showed the ESP and pH from soil samples of the western Songnen Plain (covering all the sampling points in this study) were higher than 20% and 8.5, respectively, indicating that all the soil samples exhibited intensive alkali characteristics according to the international classification standard proposed by USSLS [48]. The standard deviation of 0.915 ds/m and CV of 96.45% also showed that there was significant heterogeneity among the EC values of all the soil samples in the study. Table 2 also shows that the samples selected belong to a typical soil texture of clay loam, with the clay content of all soil samples varying in a narrow range from 25.01% to 30.99%; the standard deviation of 1.61% and CV of 5.74% also explained that the differences in clay content were not evident within the soil samples. As the soil samples were fully dried in natural conditions, the water content varied from 2.01% to 4.47%. 

**Figure 5:**
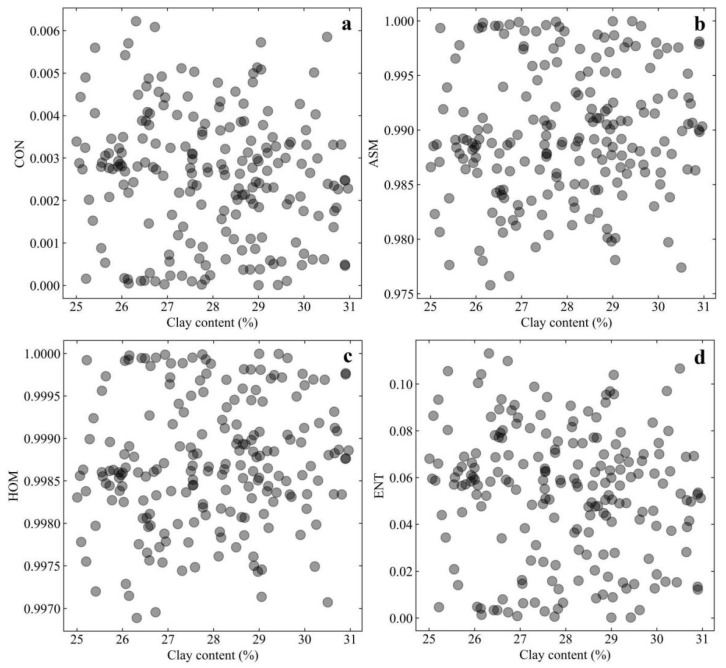
Scatter diagram between clay content and GLCM texture features. (**a**) for texture feature of CON; (**b**) for texture feature of ASM; (**c**) for texture feature of HOM; (**d**) for texture feature of ENT.

From Table 2, it also can be seen that the clay content of the soil samples in this study covered a very small range of 25.01% to 30.99%. To quantify the effects of clay content on the extent of desiccation cracks, a complementary study was carried out between the clay content and the four selected GLCM texture features (Figure 5). Notably, the scatter diagrams in Figure 5 did not show clear regularity for the data points, indicating that the clay content of soda saline–alkali soils was not sensitive to GLCM texture features.

### 3.2. Optimal Texture Features

Table 3 shows the maximum correlation coefficients between EC values and the 13 GLCM texture features in the 0°, 45°, 90°, and 135° directions under different gray levels and pixel steps. The table also indicates that the same trends appeared between each texture feature and EC values of all soil samples. Texture features such as CON, ASM, ENT, HOM, CS, CP, MP, SA, and SE had a high correlation with EC, with correlation coefficients all above 0.7, while the other four texture features, namely, COR, SV, IC1, and IC2, had poor correlation with EC values with correlation coefficients less than 0.58. Therefore, four optimal texture features (CON, ASM, ENT, and HOM) were selected in this study to analyze the ability of GLCM texture features to characterize the surface cracks of soda saline–alkali soils, considering both their high relationship with EC and their common application in texture feature analysis. Particularly, CON returns the amount of local variation in an image, reflecting the sharpness of the image and the intensity of the texture. ASM (also referred to as energy) measures the sum of squared elements in the GLCM and ranges from 0 to 1. It describes the thickness of the image texture feature and the uniformity of pixel distributions. ENT measures the randomness of the intensity distribution in the image and represents the amount of information in the image. HOM usually represents a value that measures the distribution closeness of elements in the GLCM to the GLCM diagonal and ranges from 0 to 1.

### 3.3. Analysis of GLCM Parameters

#### 3.3.1. Effects of Directions

**Figure 6:**
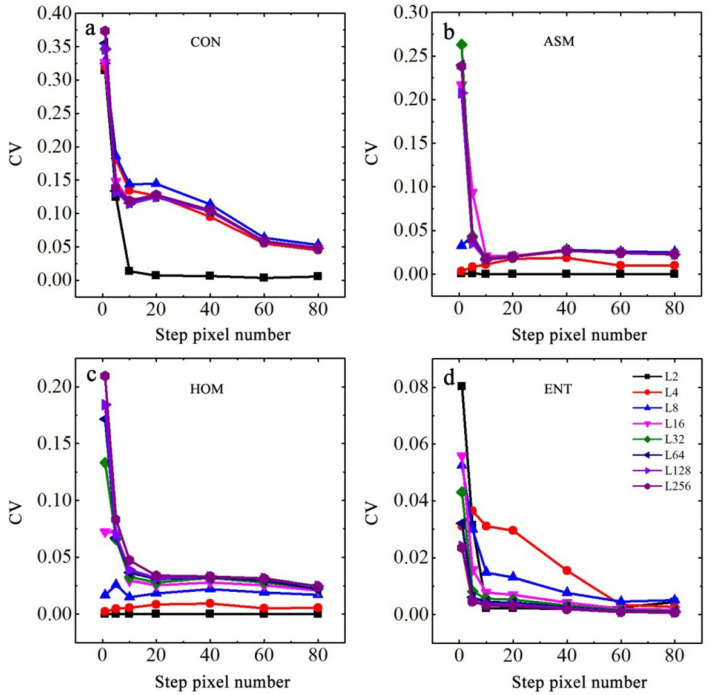
Coefficients of variation of four texture features in four directions of a typical soil sample. (**a**) for texture feature of CON; (**b**) for texture feature of ASM; (**c**) for texture feature of HOM; (**d**) for texture feature of ENT.

Figure 6 shows the coefficients of variation (CV) of CON, ASM, ENT, and HOM extracted from the 0°, 45°, 90°, and 135° directions, which were computed from the GLCMs of a typical soil sample under different gray levels and pixel steps. As shown in Figure 6a, the CV of CON in the four directions reached its highest when the step size was 1 pixel, after which the CV decreased significantly with increasing step size and gradually stabilized until it reached 60 pixels. Figure 6b shows that when the gray level was larger than 16, the CV of ASM decreased with the steps and stabilized at a step size of 10 pixels. Figure 6b also indicates that CV was no longer affected by step size when the gray level was less than 8. Figure 6c shows that the CV of HOM had the same trend as ASM but with a larger difference at various gray levels. Figure 6d shows that the CV of ENT had a similar trend with CON at the same gray level, but the difference was larger than that of CON when the step sizes were 10, 20, and 40 pixels. Therefore, there were certain differences in the extraction results of texture features computed from different directions, gray levels, and step sizes, which also explained the differences in the maximum correlation coefficients between the texture features and EC values in Table 3. To effectively consider the workload and remove the effect of direction, the mean values of texture features in the directions of 0°, 45°, 90°, and 135° were selected to further analyze the effect of texture features on the surface cracking status of soda saline–alkali soils under different gray levels and step sizes.

#### 3.3.2. Effects of Gray Levels and Step Sizes

**Figure 7:**
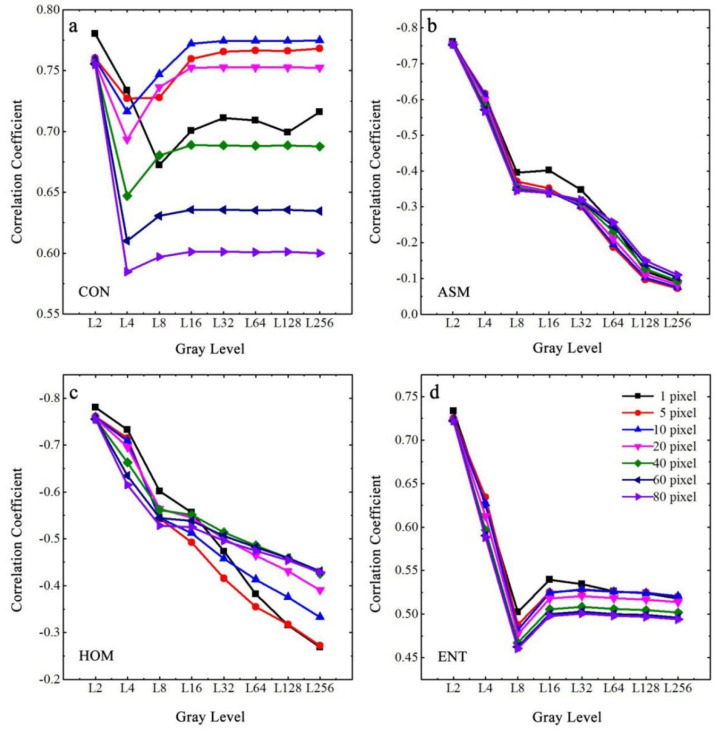
Correlation coefficients between four selected GLCM texture features and EC of the cracked soil samples. (**a**) for texture feature of CON; (**b**) for texture feature of ASM; (**c**) for texture feature of HOM; (**d**) for texture feature of ENT.

To quantify the response of GLCM texture features on the salinity of soda saline–alkali soil in the Songnen Plain of China, the mean values in four directions (0°, 45°, 90°, and 135°) were extracted under different gray levels and step sizes for all selected texture features, including CON, ASM, ENT, and HOM. Subsequently, correlation analysis was performed between the EC values and the texture features derived from different GLCM parameters, as shown in Figure 7. It can be seen from Figure 7a that the correlation coefficient between CON and EC of the soil samples was significantly positive, with an initial decreasing and then increasing trend, which finally became stable at a step size of 16 pixels. Although the lowest correlation coefficient of CON for 1 pixel in the step size occurred at a gray level of 8, the lowest values in all correlation coefficient curves under other step sizes were found at the same gray level of 4. Figure 7b shows that the correlation coefficient between the ASM and EC of the soil samples decreased with increasing gray levels, while the step size had little effect on the results of the correlation analysis. Figure 7c shows that although the trend of the correlation coefficient curves between HOM and EC were basically the same as those of ASM, a significant difference was still found under various step sizes. Figure 7d indicates that the curve shapes of the positive correlation coefficients between ENT and EC decreased until a gray level of 8, after which the correlation curves gradually increased and became stable at a gray level of 32. In addition, Figure 7 also shows that when the gray level of the crack images was determined, the correlation coefficients of the four texture features and EC values gradually decreased with increasing step size, especially when the gray level was less than 8, and the difference in the correlation coefficients also showed a more evident tendency when the gray level increased.

### 3.4. Cross-Correlation Analysis between Different Texture Features

**Figure 8:**
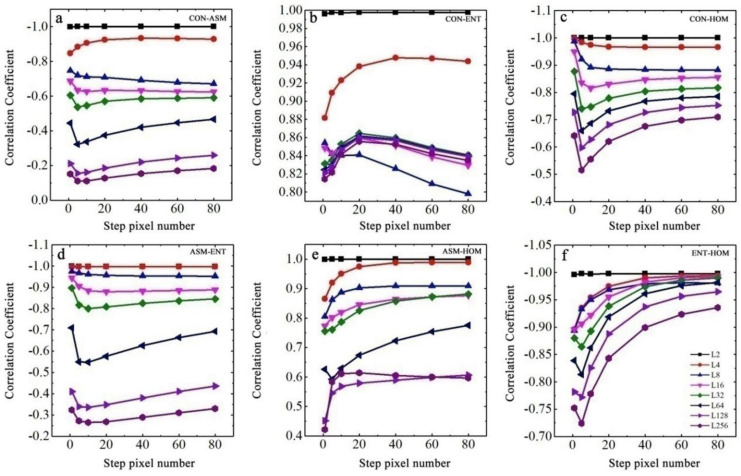
Cross−correlation coefficients of four GLCM texture features of the cracked soil samples. (**a**) between CON and ASM; (**b**) between CON and ENT; (**c**) between CON and HOM; (**d**) between ASM and ENT; (**e**) between ASM and HOM; (**f**) between ENT and HOM.

As discussed in Section 3.3, the difference in the correlation between each texture feature and EC values of soil samples was quite small, given the step size of 1 pixel and gray level of 2. As the gray level and step size increased, the difference between different texture features and the EC values of soil samples increased. Figure 8 shows the analysis results of the cross-correlation among the four selected GLCM texture features of CON, ASM, HOM, and ENT under different step sizes and gray levels. We observed that when the gray level was 2 and the step size was 1 pixel, the two different texture features were highly correlated with a correlation coefficient close to 1. However, the correlation between any two texture features rapidly decreased as the gray level increased once the step size was determined. Figure 8 also indicates that among all combinations of two texture features, the intervals of cross-correlation curves under different gray levels usually increased until the largest difference appeared at a step size of five pixels. After that, the curves of cross-correlation became close and gradually stabilized with increasing step sizes, except for the combination between CON and ENT.

### 3.5. Logarithmic Regression Models between EC and Texture Features

Table 4 shows the statistical parameters of the mean values of the four texture features in four directions of 0°, 45°, 90°, and 135° for all soil samples, which were calculated under a gray level of 2 and step size of 1 pixel. Table 4 shows that the CON extraction results had the lowest values, ranging from 4.82 × 10^−6^ to 6.2 × 10^−3^, followed by ENT varying from 1.92 × 10^−4^ to 1.13 × 10^−1^, while ASM and HOM had relatively high values ranging from 9.76 × 10^−1^ to 9.99 × 10^−1^ and from 9.97 × 10^−1^ to 9.99 × 10^−1^, respectively. In addition, the distributions of CON and HOM of different soil samples were relatively discrete, with CVs of 58.25% and 52.71%, respectively. The coefficient of variation (CV) values of 0.59% and 0.08% indicated that the extraction results of ASM and HOM texture features were relatively concentrated. Table 4 also shows that the kurtosis values of the four texture features were all less than 0, indicating that the overall distribution of the texture features was relatively flat, but the characteristic was not evident because the range was only from −0.70 to −0.61, whereas the skewness between −0.22 and 0.06 indicated that texture features of the soil samples, including CON, ASM, ENT, and HOM, basically conformed to the normal distribution.

**Figure 9:**
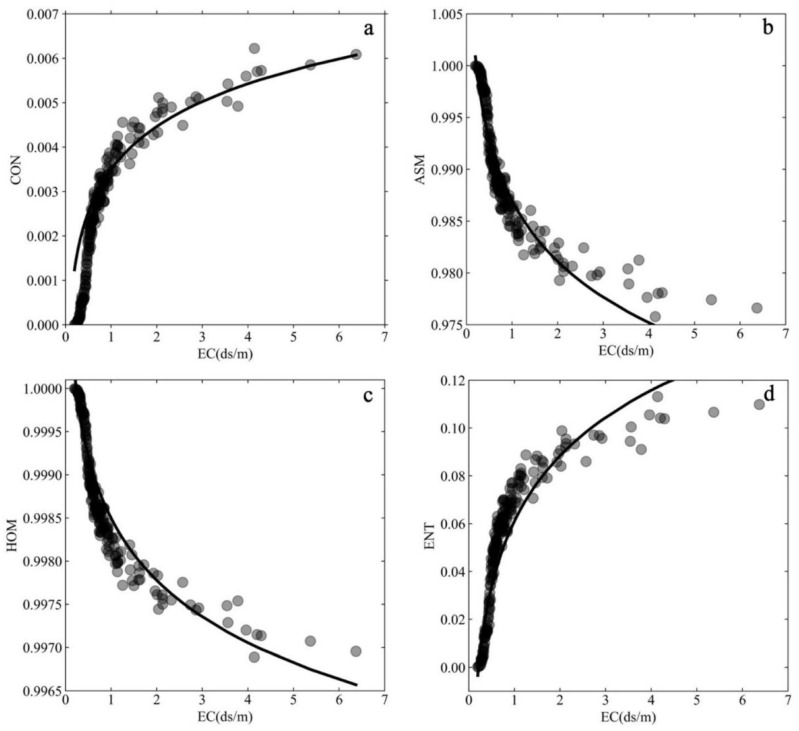
Logarithmic fitting results between EC and four GLCM texture features of the cracked soil samples. (**a**) between EC and CON; (**b**) between EC and ASM; (**c**) between EC and HOM; (**d**) between EC and ENT.

To further illustrate the response of the GLCM texture features to the salinity of soda saline–alkali soils in the Songnen Plain, regression models were developed between the EC values and the texture features of CON, ASM, ENT, and HOM of the cracked soil samples. Figure 9 shows the scatter points between the mean values in the 0°, 45°, 90°, and 135° directions of the four typical texture features mentioned above and the EC values of all cracked soil samples under the optimal GLCM parameters (gray level of 2 and step size of 1 pixel). As shown in Figure 9, all four texture features had a logarithmic relationship with the EC values of soil samples, where CON and ENT were positively correlated with EC values and ASM and HOM were inversely proportional to EC values. 

The established models in Table 5 show that both CON and HOM had the best logarithmic correlation with EC, both with R^2^ of 0.92, followed by ASM and EC with an R^2^ of 0.90. Although the weakest relationship was found between ENT and EC, R^2^ was still high, with a value of 0.88. In addition, Table 5 shows that all logarithmic models had very low RMSE, ranging only from 2.12 × 10^−4^ to 9.68 × 10^−3^, which suggested that texture features of GLCM could effectively characterize the salt content of soil samples. Thus, GLCM texture features can be considered as good indicators of the salt-determined crack status of the soda saline–alkali soil.

## 4. Discussion

From Table 2, it can be seen that the clay content did not play an important role in desiccation cracking of soda saline–alkali soils in this study. This was because the original minerals of soda saline–alkali soils in the Songnen Plain are quartz and feldspar, and the secondary mineral relates to illite/smectite formation with an interlayer ratio above 0.5 according to the results of X-ray diffraction complete analysis measured by Zhang et al. [49] and Wang et al. [50]. This result indicates that the clay content and clay mineral composition did not play important roles in the shrinkage and cracking of the soil samples because of the activity index covering a very low range from 0.33 to 0.48. The soil salinity therefore can be considered as the main role in the desiccation cracking process of salt-affected in this study. Yang et al. [51] found that the soil salinity was highly correlated with both ESP and SAR of the salinized soils in Songnen Plain since the main salt minerals were NaHCO_3_ and Na_2_CO_3_, which made Na^+^ the dominant exchangeable cation; their results also indicated that this kind of salt mineral composition leads to an alkalinization reaction and thus disperses the cementation between the clay soil particles. In addition, according to the measurements results of different ions, Chi and Wang [52] found that Na^+^ occupies an absolute dominant role in the cations of the saline–alkali soils in the Songnen Plain with content much larger than those of the cations including K^+^, Ca^2+^, and Mg^2+^, indicating that Na^+^ affects the desiccation cracking of soda saline–alkali soils in Songnen Plain, China, to a certain extent. Specifically, the previous studies from Zhang et al. [53] and Yu et al. [54] turned out that a kind of thick bound water film can be found forming around the soil particles, which is caused by the interaction between colloidal particles and adsorbent cations (especially for Na^+^ with a hydrolysis radius compared with other cations within the type of soda saline–alkali soils in Songnen Plain). This kind of water film always in turn reduces the cohesion between soil particles and then results in a decrease in the soil strength [55]. Aksenov et al. [56] also found that the combined water film generated among soil particles could reduce the internal friction angle and shear strength of salinized soil samples, thus making the surface of saline–alkali soils more prone to shrinkage and cracking. Moreover, many studies have indicated that the diffuse double layer (DDL) also plays an important role in determining the shrinking and cracking processes of saline soils during water evaporation [57,58]. Specifically, water evaporation causes thinning of the DDL and a reduction in the distance between soil particles, which results in the propagation of desiccation cracks on the soil surface. Therefore, a higher salt content makes soil particles combine more tightly, which is manifested by more soil volume shrinkage and more complex soil cracking.

The selection of crack parameters usually shows great effects on the quantification of soil surface cracks. Although many previous studies have shown that geometric parameters (such as crack length, crack area, crack length density, and crack area density) have been commonly used, they still have certain defects in quantitatively characterizing the surface cracks compared with texture features. Specifically, crack length and crack area cannot show convergence [59,60], while crack length density and crack area density are unable to adequately describe the distributions of desiccation cracks generated from the random cracking locations of the soil samples with low repeatability [61,62]. In addition, the crack densities mentioned above also fail to provide an idea of the propagation of soil cracks in different directions [63]. Due to its strong self-correlation under natural conditions, GLCM texture analysis can thus offer an easy way to describe the cracking status of the soil surface quantitatively and effectively. 

Under natural conditions, there are differences in the extraction results of GLCM texture features in various directions, including 0°, 45°, 90°, and 135°, which may be related to the presence of slight slopes on certain soil surfaces that cause the water to flow in a specific direction after precipitation. However, the difference in texture features of field soil samples due to the direction is not distinct because most of the process and direction of crack development are random. Moreover, the direction of the crack patterns of soil samples may not be accurately obtained in the photo process in the field, and there may be operations, such as rotation of the images in the post-processing stage, which make it difficult to ensure the same standard direction of the texture features for all soil samples. The characterization ability of GLCM texture features on the EC values generally showed a decreasing trend with the increasing step size. This was because when the step size is small, the extraction results of the texture features can effectively distinguish the grayscale of the crack region. However, when the step size is larger than the width of the soil crack, the calculation results of the GLCM texture features ignore the information of some crack regions, resulting in a decrease in the correlation with soil EC. Further, the increasing gray level reduced the correlation between texture features and soil salinity, which may be because when the gray level is relatively low, the gray value of the crack region is more distinct from the gray level of other surface areas of the soil samples. In contrast, with an increasing gray level, the uncertainty of the pixel distribution is enhanced, and the consistency of gray values is weakened [64,65,66], which rapidly decreases the ability of ASM and HOM to characterize crack conditions. In addition, although the amount of local variation in the cracked patterns of soil samples was the most notable in the lowest image information when the gray level was set to 2 (all the images only represent crack and non-crack regions), increasing the gray level gradually reduced the proportion of gray levels within the crack regions and thus made CON (returns the local variation of crack images) and ENT (returns the randomness and complexity of crack images) decrease and gradually become stable. Therefore, GLCM texture features were the best for characterizing the surface cracking status of soda saline–alkali soils under the optimal gray level of 2 and step size of 1 pixel.

From Section 3.5, it can be seen that the EC values of soda saline–alkali soils showed clear logarithmic relationships with different GLCM texture features, indicating that a new online measurement method of soil salinity can therefore be proposed using regression models based on the GLCM texture features, which are computed with optimal GLCM computing parameters. Specially, the procedures can be described as follows: firstly, taking crack patterns under field conditions using a unified photographic standard; secondly, performing geometric correction and preprocessing operations on all crack images; after that, extracting GLCM texture features under optimal computing parameters including gray level, direction, and step size; subsequently, developing regression models with a certain number of soil samples; thereafter, importing the texture features extracted from all other soil samples into the best regression model to predict the EC values of soil samples; and finally, calibrating all estimated EC values using the measured ones in laboratory for better accuracy. In practical applications, this potential method can thus be selected to measure the soil salinity non-destructively, effectively, and accurately. In addition, this kind of method can be further extended to aerial remote sensing for simultaneous measurement of soil salinity in a large range, which can provide important guidance for ecological restoration, agricultural production, and engineering construction in saline–alkali areas.

## 5. Conclusions

In this study, quantitative analyses were conducted to study the effects of GLCM computing parameters on the relationship between different texture features and the EC values of soda saline–alkali soil samples. Although the texture features in different directions were different, their influence was quite limited; as the gray level and step size increased, the correlation between texture features and soil salinity greatly decreased. Although the cross-correlation between various texture features decreased rapidly with the increase in gray level, it was weakly affected by step size. The soil salinity of the soda saline–alkali soils in the Songnen Plain was correlated with the surface crack status, indicating that they can be determined by the GLCM texture features. In further studies, aerial remote sensing for simultaneous prediction of soil salinity in a large range are required to provide guidance for ecological restoration, agricultural production, and engineering construction.

## Figures and Tables

**Table 1:** GLCM texture feature calculation formulas.

Texture Features	Formulas
**Contrast (CON)**	$CON=\overset{Ng-1}{\underset{n}{\sum }}n^{2}\left\{\overset{Ng}{\underset{i=1}{\sum }}\overset{Ng}{\underset{j=1}{\sum }}p\left(i,j\right)\right\},\left|i-j\right|=n$
**Angular second moment (ASM)**	$\mathrm{ASM}=\underset{I}{\sum }\underset{J}{\sum }{\left\{P\left(i,j\right)\right\}}^{2}$
**Entropy (ENT)**	$ENT=-\overset{Ng}{\underset{i=1}{\sum }}\overset{Ng}{\underset{j=1}{\sum }}p\left(i,j\right)\log\left(p\left(i,j\right)\right)$
**Homogeneity (HOM)**	$HOM=\overset{Ng}{\underset{i=1}{\sum }}\overset{Ng}{\underset{j=1}{\sum }}\frac{1}{1+{\left(i-j\right)}^{2}}p\left(i,j\right)$
**Correlation (COR)**	$COR=\frac{\sum_{i=1}^{Ng}\sum_{j=1}^{Ng}\left(ij\right)p\left(i,j\right)-u_{x}u_{y}}{\sigma_{x}\sigma_{y}}$
**Cluster shade (CS)**	$CS=\overset{Ng}{\underset{i=1}{\sum }}\overset{Ng}{\underset{j=1}{\sum }}{\left(\left(i-u_{i}\right)+\left(j-u_{j}\right)\right)}^{3}p\left(i,j\right)$
**Cluster prominence (CP)**	$CP=\overset{Ng}{\underset{i=1}{\sum }}\overset{Ng}{\underset{j=1}{\sum }}{\left(\left(i-u_{i}\right)+\left(j-u_{j}\right)\right)}^{4}p\left(i,j\right)$
**Max probability (MP)**	MP=max{p(i,j)}
**Sum average (SA)**	$SA=\overset{2Ng}{\underset{1=2}{\sum }}ip_{x+y}\left(i\right)$
**Sum entropy (SE)**	$SE=-\overset{2Ng}{\underset{1=2}{\sum }}p_{x+y}\left(i\right)\log\left\{p_{x+y}\left(i\right)\right\}$
**Sum variance (SV)**	$SV=\overset{2Ng}{\underset{1=2}{\sum }}{\left(i-SumEntropy\right)}^{2}p_{x+y}\left(i\right)$
**Information of correlation (IC1)**	$IC1=\frac{HXY-HXY1}{max\left(HX,HY\right)}$
**Information of correlation (IC2)**	IC2=(1−exp[−2*(HXY2−HXY)])12

**Table 2:** Statistical description of soil properties of the soil samples.

Parameters	Min	Max	Mean	Standard	CV%	Skewness	Kurtosis
**EC (ds/m)**	0.20	6.37	0.95	0.915	96.45	3.04	10.9
**pH**	8.55	11.16	10.06	0.53	5.36	0.34	−0.85
**Moisture (%)**	2.01	4.47	2.95	0.58	19.32	−1.14	0.06
**Clay (%)**	25.01	30.99	27.88	1.61	5.74	−1.01	0.06
**Silt (%)**	30.06	41.95	35.98	3.51	9.77	−1.30	0.02
**Sand (%)**	28.19	39.38	33.86	3.39	10.01	−1.13	0.13

N = 200; CV, coefficient of variation.

**Table 3:** Maximum correlation coefficient between texture features and EC in four directions under different gray levels and step sizes.

Texture Features	0°	45°	90°	135°
**CON**	0.82	0.78	0.76	0.78
**ASM**	−0.77	−0.76	−0.75	−0.76
**ENT**	0.74	0.73	0.72	0.73
**HOM**	−0.82	−0.78	−0.76	−0.78
**COR**	−0.47	−0.29	−0.31	−0.39
**CS**	−0.75	−0.75	−0.76	−0.75
**CP**	0.75	0.76	0.77	0.76
**MP**	−0.77	−0.76	−0.76	−0.76
**SA**	0.74	0.73	0.72	0.73
**SE**	−0.74	−0.73	−0.72	−0.73
**SV**	0.37	0.44	0.37	0.34
**IC1**	0.57	0.58	0.61	0.58
**IC2**	−0.31	−0.43	−0.31	−0.43

N = 200; significance level α = 0.05; CON, contrast; ASM, angular second moment; ENT, entropy; HOM, homogeneity; COR, correlation; CS, cluster shade; CP, cluster prominence; MP, max probability; SA, sum average; SE, sum entropy; SV, sum variance; IC1 and IC2, information of correlation based on different equations.

**Table 4:** Statistical description of four GLCM texture features of cracked soil samples.

Parameters	Min	Max	Mean	Standard	CV%	Skewness	Kurtosis
**CON**	4.82 × 10^−6^	6.2 × 10^−3^	2.6 × 10^−3^	1.5 × 10^−3^	58.25	0.06	−0.61
**ASM**	9.76 × 10^−1^	9.99 × 10^−1^	9.89 × 10^−1^	5.9 × 10^−3^	0.59	0.02	−0.69
**ENT**	1.92 × 10^−4^	1.13 × 10^−1^	5.29 × 10^−2^	2.79 × 10^−2^	52.71	−0.22	−0.70
**HOM**	9.97 × 10^−1^	9.99 × 10^−1^	9.98 × 10^−1^	7.1 × 10^−4^	0.08	−0.06	−0.61

N = 200; CON, contrast; ASM, angular second moment; ENT, entropy; HOM, homogeneity; CV, coefficient of variation.

**Table 5:** Logarithmic regression models based on EC and texture features of soda saline–alkali soil samples with surface cracks.

Texture Features	Logarithmic Regression Models	R^2^	RMSE
**CON**	y = 0.0032 × lg(x) + 0.005	0.92	4.24 × 10^−4^
**ASM**	y = −0.0196 × lg(x) + 0.987	0.90	1.86 × 10^−3^
**ENT**	y = 0.091 × lg(x) + 0.065	0.88	9.68 × 10^−3^
**HOM**	y = −0.0024 × lg(x) + 0.998	0.92	2.12 × 10^−4^

RMSE, root mean square error; $\mathrm{RMSE}=\sqrt{\frac{\sum_{i=1}^{n}{\left(\;y\;\prime -y\right)}^{2}}{n}}$, n represents the number of soil samples, y stands for the measured texture feature, and y’ stands for the observed texture feature based on models; CON, contrast; ASM, angular second moment; ENT, entropy; HOM, homogeneity.

## Data Availability

Not applicable.
